# Improved control on the morphology and LSPR properties of plasmonic Pt NPs through enhanced solid state dewetting by using a sacrificial indium layer†

**DOI:** 10.1039/c8ra09049a

**Published:** 2019-01-17

**Authors:** Sundar Kunwar, Mao Sui, Puran Pandey, Zenan Gu, Sanchaya Pandit, Jihoon Lee

**Affiliations:** Department of Electronic Engineering, College of Electronics and Information, Kwangwoon University Nowon-gu Seoul 01897 South Korea jihoonlee@kw.ac.kr

## Abstract

Platinum (Pt) nanoparticles (NPs) are important nano-material components in various catalytic, photonic and electronic applications, yet face challenges in the fabrication of desired morphology and uniformity with the conventional solid-state dewetting approach. Specifically, the necessity of high annealing temperatures, typically above 800 °C due to the low diffusivity of Pt atoms, limits the morphological and functional tunability of Pt NPs. In this work, the fabrication of Pt NPs with an improved configuration, spacing and uniformity is demonstrated through the enhancement of solid state dewetting by using a sacrificial indium (In) layer on sapphire (0001). The well-defined Pt NPs demonstrate the dynamic localized surface plasmon (LSPR) bands in the visible range between ∼400 and 700 nm depending on the size and spacing of NPs. The LSPR peak intensity and width are also varied depending on the uniformity of Pt NPs. The overall dewetting magnitude is significantly enhanced through the inter-mixing of In and Pt atoms at the In/Pt interface that eventually results in the formation of an In–Pt alloy. During the dewetting process the In atoms desorb from the NP matrix by atomic sublimation, which gives rise to pure Pt NP fabrication. In sharp contrast to the pure Pt film dewetting, the Pt NPs in this approach demonstrate significantly improved spatial arrangement with well-defined configuration and uniformity. In addition, the ratio of In can be readily controlled along with the thickness of the Pt layer to alter the dewetting kinetics and thereby the surface morphology of Pt NPs. Specifically, large hexagonal, semi-spherical and small hexagonal Pt NPs are obtained through the dewetting of In_75 nm_/Pt_25 nm_, In_20 nm_/Pt_20 nm_ and In_2.5 nm_/Pt_7.5 nm_ bilayers respectively.

## Abstract 2

Fabrication of Pt NPs with the improved configuration, spacing, uniformity and localized surface plasmon resonance (LSPR) response is demonstrated.

## Graphical abstract

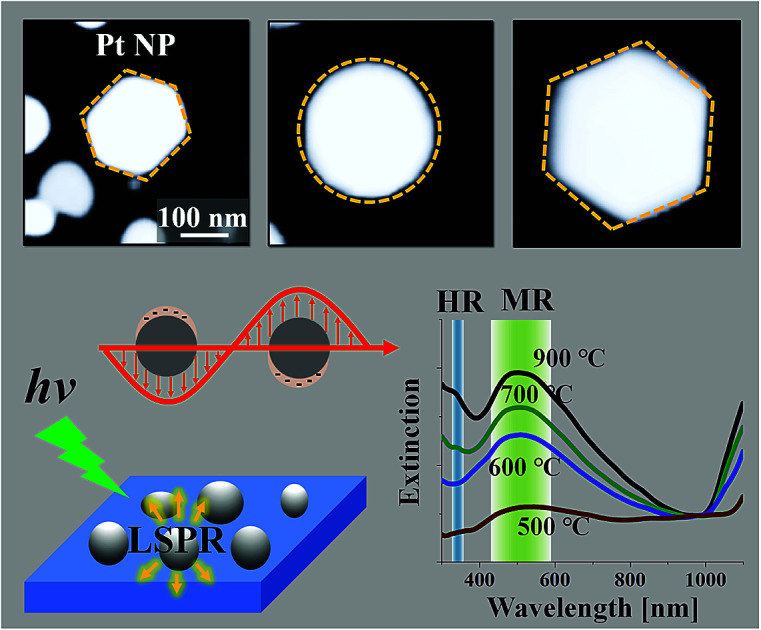

## Introduction

In recent years, metallic nanostructures have gained tremendous research attention due to their ample optical, electrical, catalytic and magnetic properties for a wide range of applications in optoelectronics, photonics, catalysis, sensing and biomedical fields.^1–5^ One of the most attractive features of metallic nanoparticles (NPs) is the localized surface plasmon resonance (LSPR), which refers to the excitation of collective electron oscillation confined on the surface of metallic NPs by the photon incidence. The LSPR effect of metallic NPs can be utilized to enhance light absorption, charge separation, chemical reactivity and target sensitivity of corresponding devices.^6–11^ As an example, the power conversion efficiency of dye-sensitized solar cells can be significantly improved by the increased photocurrent density due to LSPR absorption by the incorporation of NPs.^7^ Furthermore, the position, intensity and bandwidth of LSPR can be inherently modulated by control of the size, shape and density of plasmonic metallic NPs, which enables the appropriate tuning of various plasmonic NP properties such as in optical, electrical, catalytic, magnetic and sensing applications.^12–14^ At the same time, the Pt NPs exhibit a distinct LSPR band, chemical durability and corrosion resistance, high hydrogen affinity and strong interaction with the supporting materials, which makes them promising candidates for various catalytic, optoelectronic and sensing applications.^15–20^ However, the fabrication of well-defined and properly spaced Pt NPs using the dewetting of metal thin films has been challenging due to the high surface energy and low diffusivity of Pt atoms at normal growth conditions below 800 °C.^21–23^ The fabrication of well-defined Pt NPs and the exploration of their LSPR properties can be an important reference for the associated applications. In this work, the fabrication of Pt NPs of well-defined configurations, spatial arrangement and uniformity are demonstrated on sapphire (0001) based on the enhanced solid state dewetting by using a sacrificial In layer between Pt film and sapphire. The study on the LSPR properties of Pt NPs shows the formation of dynamic LSPR bands in the visible wavelength whose spectral position, intensity and shape are readily tuned by the variation of size, shape and spacing of Pt NPs. The overall dewetting process is facilitated by the intermixing between In and Pt atoms and formation of In–Pt alloy, which significantly enhances the global diffusion of atoms. Along with the annealing, the dewetting of In–Pt alloy nanostructures and subsequent sublimation of In atoms give a arise to the formation of Pt NPs. By controlling the ratio of In and Pt, various size, density, spacing and configuration of Pt NPs are demonstrated.

## Experimental details

### Sample preparation and Pt NP fabrication

In this study, 430 μm-thick double-side polished *c*-plane sapphire wafers with ± 0.1° off-axis (iNexus Inc., South Korea) were utilized for the fabrication of nanostructures. Prior to the growth, wafers were diced into an appropriate size and degassed in a pulsed laser deposition (PLD) chamber under 1.0 × 10^−4^ torr at 600 °C for 30 min. This ensures the removal of trapped particulates, oxides and water vapors from the substrates. After degassing, the bare sapphire was examined in ambient by atomic force microscope (AFM) scanning, which revealed the smooth surface morphology with less than ± 0.25 nm surface modulation as shown in Fig. S1(a).† Furthermore, the reflectance and transmittance spectra exhibited uniform spectra over 300 to 1100 nm range as displayed in Fig. S1(c) and (d)† respectively. In the next step, the In and Pt films were sequentially deposited from the respective target of In and Pt with 99.999% purity. Both metallic films were deposited under an identical condition: *i.e.* the base pressure, ionization current and rate of deposition were 1 × 10^−1^ torr, 3 mA and 0.05 nm s^−1^ respectively. The thickness of metallic film was controlled by the deposition duration, *i.e.* 20 s corresponds to 1 nm. Three distinct bilayer compositions were prepared to examine the effect of In/Pt bilayer composition on the dewetting process and thereby the evolution of various nanostructures. In the first series, a total of 10 nm thick bilayers consisting of 2.5 nm In and 7.5 nm were prepared, named as In_2.5 nm_/Pt_7.5 nm_. Similarly, for the second and third series of samples, In_20 nm_/Pt_20 nm_ and In_75 nm_/Pt_25 nm_ bilayers were deposited respectively. After the deposition of each In/Pt bilayer, the surface morphology was investigated by atomic force microscope (AFM) scanning as shown in Fig. S2(a)–(c).† The corresponding roughness (*R*_q_), surface area ratio (SAR) and cross-sectional line profiles showed that the surface modulation was gradually increased with the bilayer thickness. Then, the deposited samples were mounted on an Inconel holder and transferred to the PLD chamber for the annealing. The base pressure of annealing chamber was below 1 × 10^−4^ torr and the pre-specified temperatures between 400 and 800 °C were reached by a ramp rate of 4 °C s^−1^. At each target temperature, 450 s of dwelling duration was allocated for the matured growth surface nanostructures. The overall annealing process was computer controlled for the consistency. To finish the growth, the heating system was turned off and samples were kept under the same vaccum until the temperature was dropped to an ambient over the course of time.

### Pt NP characterization

After the fabrication, the surface morphologies were characterized by an AFM under a non-contact mode (XE-70, Park Systems, United States of America) in ambient. The same batch of AFM probes with a typical tip height of ∼17 μm and radius of <10 nm was used. Furthermore, a large scale analysis of surface morphologies was pergored by using a scanning electron microscope (SEM) (CX-200, COXEM, South Korea). The elemental analysis was performed by using an energy-dispersive X-ray spectroscope (EDS) (Noran System 7, Thermo Fisher, United States of America). For the optical characterization, UNNIRAM II system (UniNanoTech, South Korea) equipped with a CCD detector, ANDOR sr500 spectrograph and halogen and deuterium light source was utilized. The reflectance and transmittance spectra were obtained in a normal incidence mode at an ambient condition as illustrated in Fig. S1(e).†

## Results and discussion

**Fig. 1:**
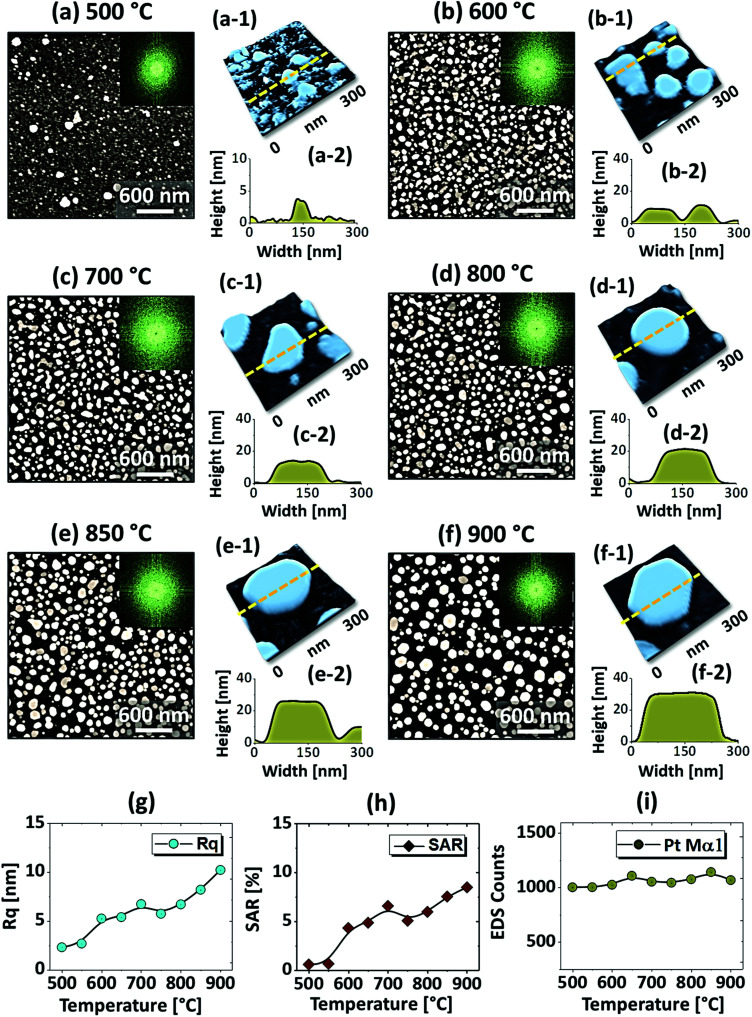
Evolution of Pt NPs from In_2.5 nm_/Pt_7.5 nm_ bilayers based on annealing between 600 and 900 °C for 450 s. (a)–(f) AFM top-views (3 × 3 μm^2^) of Pt NPs. Insets show the Fourier filter transform (FFT) power spectra of the corresponding AFM top-views. (a-1)–(f-1) Enlarged AFM 3D side-views of typical Pt NPs at different temperature. (a-2)–(f-2) Corresponding line profiles of AFM side-views. (g)–(i) Summary plots of RMS roughness (*R*_q_), surface area ratio (SAR) and energy dispersive X-ray spectroscope (EDS) counts, corresponding to the Pt Mα1 peak at 2.047 keV.

Fig. 1 shows the evolution of compact to widely spaced hexagonal Pt NPs on sapphire (0001) from the In_2.5 nm_/Pt_7.5 nm_ bilayer annealed between 500 and 900 °C for 450 s. As represented by the AFM top-views, enlarged side-views and cross-sectional line profiles, the diverse configuration, size and density such as the slight-elongated, semi-spherical and widely-spaced hexagonal Pt NPs were obtained along with the control of annealing temperature. Generally, the growth of isolated Pt NPs from the continuous In/Pt bilayer can be described based on the temperature driven solid-state dewetting mechanism.^24^ The dewetting kinetics can be controlled by altering the growth parameters such as temperature, pressure, annealing duration and film thickness as well as the intrinsic properties of films and substrates such as diffusivity, surface energy, interface energy and crystal structures.^24–26^ On top of these parameters, in this work, the overall dewetting process of Pt film on sapphire has been readily altered or enhanced by the introduction of In layer. The In was chosen as an intermediate layer between Pt film and sapphire because of its low surface energy, high surface diffusivity as well as low sublimation temperature.^27^ On the other hand, the Pt has a high surface energy and low surface diffusivity and hence exerts robust thermal stability against the dewetting, which can be more significant at low annealing temperature range.^27,28^ During the annealing, the In atoms can be activated and thus begin to diffuse at the In/Pt bilayer interface even at relatively lower temperature. As a consequence, the incorporation of In atoms can occur at the interface, which can give a rise to the inter-mixing process.^29^ Due to the intermixing between In and Pt atoms, the formation of In–Pt alloy system can be expected and therefore, the global diffusion can be significantly enhanced. Eventually, the In–Pt bimetallic nanostructures can be nucleated based upon the temperature of system. Meanwhile, due to the high vapor pressure of In, the In atoms can sublimate around ∼360 °C and the rate of In sublimation exponentially increases with the temperature such that the vapor pressure can reach ∼2 × 10^−6^ torr at 600 °C and ∼5 × 10^−4^ torr at 800 °C. On the other hand, due to the intermixing of In and Pt atoms, the lower temperature eutectic can occur with the alloy, which can result in the enhanced In sublimation at much lower temperature.^29^ Therefore, the In atoms can gradually sublimate along with the temperature and as a result, the near pure Pt NPs with distinct configuration and structure can be formed by the concurrent effect of dewetting and sublimation.^30^ In comparison with the conventional dewetting of pure Pt on sapphire, the In component accelerates the overall dewetting process and hence the well-structured and isolated Pt NPs can be developed.^31,32^ In the case of pure Pt, only minor surface roughness development with the formation of hillocks and pinholes was observed below 600 °C and the Pt NPs were compact and irregular even at 900 °C because of the low surface diffusivity of Pt atoms as discussed.^31,32^ Apparently, in this work, the void nucleation, growth and NP formation was obtained at relatively lower temperature and the Pt NPs were regular and well-separated as compared with the pure Pt dewetting, which can be correlated to the dewetting enhancement by the sacrificial In component as mentioned. As shown in Fig. 1(a), at 500 °C, the dewetting process was initiated with the formation of tiny pinholes and overgrown NPs of ∼5 nm height and ∼40 nm diameter. Since the substrate was not exposed at this temperature, the pinholes might not be completely penetrated through the film to reach the substrate. The typical Pt nanostructures at specific temperature are displayed in the corresponding 3D side-views and the dimensions are extracted by their line profiles as displayed in Fig. 1(a-1) and (a-2) respectively. Furthermore, the 2D Fourier filter transform (FFT) power spectra were presented in the insets to show the surface periodicities or the height fluctuations. For the undewetted layered structure the FFT pattern was small because of the low surface periodicities. At 600 °C, the surface morphology was drastically evolved as the layered structures were transformed into the isolated Pt NPs of 10 nm height and 100 nm diameter as shown in Fig. 1(b). The corresponding FFT pattern was slightly enlarged due to the formation of isolated NPs, which could increase surface height fluctuations. This process can be correlated to the rapid void nucleation and growth due to the enhanced diffusion of atoms and sublimation of In. By increasing the temperature between 700 and 850 °C, the transformation from irregular to semi-spherical configurations of Pt NPs was witnessed as displayed in Fig. 1(c)–(e). Meanwhile, the average height, diameter and inter particle spacing were significantly increased to ∼30, 150 and 170 nm respectively while the areal density was gradually decreased. During the self-assembly of Pt atoms, multiple factors such as surface diffusion, critical radius of voids, contact angle and equilibrium energy can affect the size and shape evolution of Pt NPs.^33^ Due to the adequate diffusion energy of atoms at high temperature, the Pt NPs can reconstruct their shape towards the lowest possible surface and interface energy and thereby become thermodynamically stable. Finally, at 900 °C, mostly hexagonal Pt NPs of average height ∼30 nm and diameter ∼200 nm were observed as shown in Fig. 1(f) due to the more favorable diffusion and coalescence of neighboring Pt NPs. The uniformity of the Pt NPs was found to be improved as the denoted by the reduced size of FFT pattern. The hexagonal Pt NPs can be originated because of the hexagonal crystallographic structure of the underlying *c*-plane sapphire.^34^ On the other hand, the top of Pt NPs became flat or truncated, which can be due to the formation of lower surface energy planes.^35^ Furthermore, the surface morphology was investigated in terms of *R*_q_ and SAR of large size AFM images. As summarized in Fig. 1(g) and (h), both the *R*_q_ and SAR were gradually increased as a function of temperatures indicating the increased average surface roughness and surface area of Pt NPs. In specific, the *R*_q_ was increased from ∼2 to 12 nm whereas the SAR from ∼1 to 9% when the temperature was varied between 500 and 900 °C. The elemental analysis was performed by the EDS spectra measurement, which showed the similar Pt counts as shown in Fig. 1(i), indicating the similar amount of Pt in each sample. And, the In peak was not observed in all samples as the In atoms can be sublimated as discussed. The specific EDS spectra for two samples at 550 and 900 °C are shown in Fig. S4,† which shows the O Kα and Al Kα peaks of the sapphire and Pt Mα1 of the NPs. This ensures that the NPs obtained in this work were nearly pure Pt NPs.

**Fig. 2:**
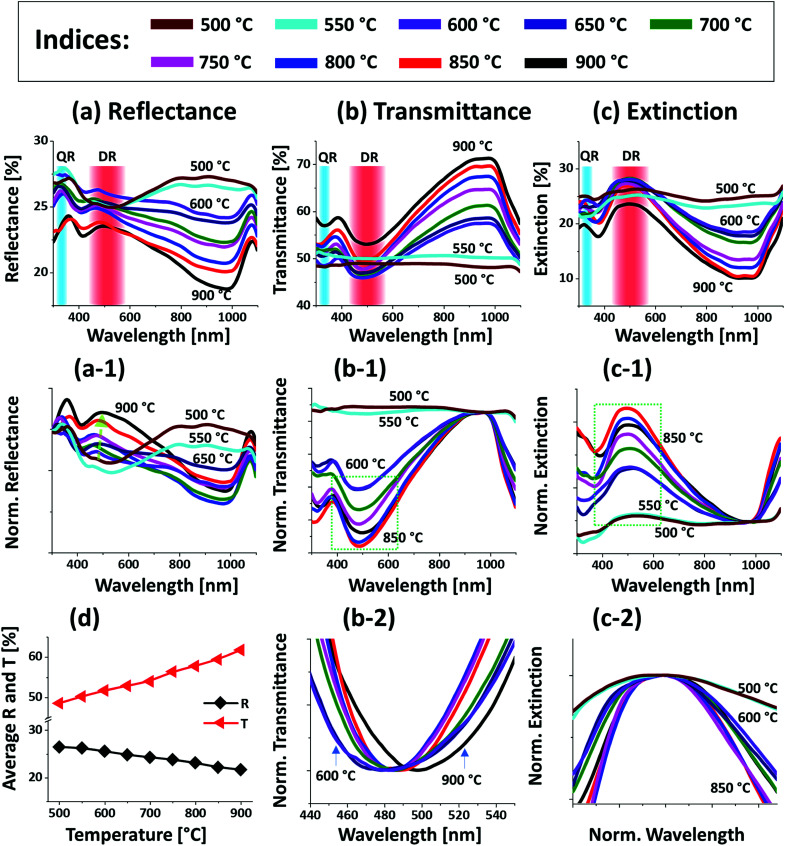
Optical properties of the Pt NPs fabricated at various temperature with the In_2.5 nm_/Pt_7.5 nm_ bilayers. (a)–(c) Reflectance (*R*), transmittance (*T*) and extinction (*E*) spectra of the Pt NPs. The formation of dipolar resonance (DR) and quadrupolar resonance (QR) modes of Pt NPs are indicated by the color bands in the (a)–(c). (a-1) Reflectance spectra normalized at 300 nm. (b-1) Normalized transmittance spectra. (b-2) Enlarged dip region of transmittance spectra at ∼490 nm, boxed region in (b-1). (c-1) Normalized extinction spectra. (c-2) Enlarged peak of extinction spectra, boxed region in (c-2). (d) Summary plot of *R* and *T*.

Fig. 2 presents the optical properties of small Pt NPs on sapphire based on the UV-VIS-NIR reflectance, transmittance and extinction spectra. In which the reflectance and transmittance spectra were experimentally measured with the normal incidence of light as shown in Fig. S1(b)† and the extinction spectra were extracted by the following relation: reflectance (%) + transmittance (%) + extinction (%) = 100%. All these optical spectra exhibited distinctive wavelength dependent characteristics based on the surface morphology of Pt NPs at various temperatures. In specific, the reflectance spectra in Fig. 2(a) exhibits a wide dip in the visible region (400–600 nm) and shoulder in the NIR region (above 750 nm) with the layered structure up to 550 °C. When the isolated Pt NPs were formed above 600 °C, a sharp transition in the reflectance spectra was observed *i.e.* the formation of UV (∼330 nm) and VIS region (∼500 nm) shoulder and dip in NIR region. For the typical Pt NPs of average size less than 200 nm, the excitation of dipolar and quadrupolar dipolar resonance mode can be expected upon light incidence.^36,37^ Specifically, the quadrupolar and dipolar resonance modes lie in the UV and VIS wavelength of electromagnetic spectrum respectively.^37^ At the same time, due to the strong backscattering with the dipolar resonance of Pt NPs, the reflectance spectra can show a shoulder development or distorted peak in the shorter wavelength as observed in Fig. 2(a). In the case of longer wavelength in NIR region, which is far from the resonance wavelength, the reflectance was high with the layered structure and it was gradually decreased with the formation of isolated Pt NPs. To reflect the effect of surface morphology of Pt NPs on optical properties, the original reflectance spectra were normalized as shown in Fig. 2(a-1). From the normalized reflectance spectra, it can be seen that the UV-VIS region exhibited the enhanced shoulder development for the isolated samples at high temperatures, which can be correlated to the enhanced backscattering effect with the clear formation of Pt NPs.^38^ In addition, the transmittance spectra in Fig. 2(b) clearly demonstrated the formation of dips in the UV at ∼330 nm and VIS region at ∼500 nm, corresponding to the quadrupolar and dipolar resonance modes with the definite Pt NPs whereas nearly flat behavior with the layered structures.^36,37^ In the NIR region, the transmittance spectra again suggested the strong transmittance with high peaks at the off-resonance wavelength range. By considering both the reflectance and transmittance, the decrease in the NIR reflectance or increased NIR transmittance with the formation of isolated Pt NPs can be due to the transition in the refractive index between air and sapphire.^39^ With the normalized transmittance spectra shown in Fig. 2(b-1), it can be observed that the UV-VIS region dips were gradually developed with the formation of more isolated and larger Pt NPs, which was enhanced with the size increment of Pt NPs, likely due to the improved LSPR absorption in the UV-VIS wavelength.^36,37^ The transmittance dips were further investigated by renormalization to 1 as shown in Fig. 2(b-2), which showed the gradual red shift from ∼480 to 500 nm along with the increased size of Pt NPs between 600 and 900 °C.^40^ The overall LSPR effect of the Pt NPs can be observed in the extinction spectra in Fig. 2(c), which clearly exhibits the quadrupolar and dipolar resonance peaks in the UV and VIS region respectively as indicated with color bands. Specifically, the intensity of the LSPR peak was increased along with the formation of larger Pt NPs at higher temperature.^41^ The resonance peaks were wider when the NP size distribution was broader at low temperature. With the improved size and shape uniformity at higher temperature, the LSPR peaks became gradually narrowed as shown in Fig. 2(c-2).^42^ From all the optical spectra, it was clearly observed that the dipolar resonance peak was much stronger and reflect the morphology changes of Pt NPs very sensitively. Furthermore, it was also found that the LSPR excitation mostly occurred in the UV-visible region likely due to the quadrupolar and dipolar resonance respectively.^36,37^ In case of average reflectance and transmittance, they showed opposite trend *i.e.* reflectance was decreased whereas transmittance was increased with the reduced surface coverage of Pt NPs as presented in Fig. 2(d). The average reflectance and transmittance values were provided to show overall quantitative reflectance and transmittance throughout the wavelength range between 300 and 1100 nm in relation to the NP surface coverage.

**Fig. 3:**
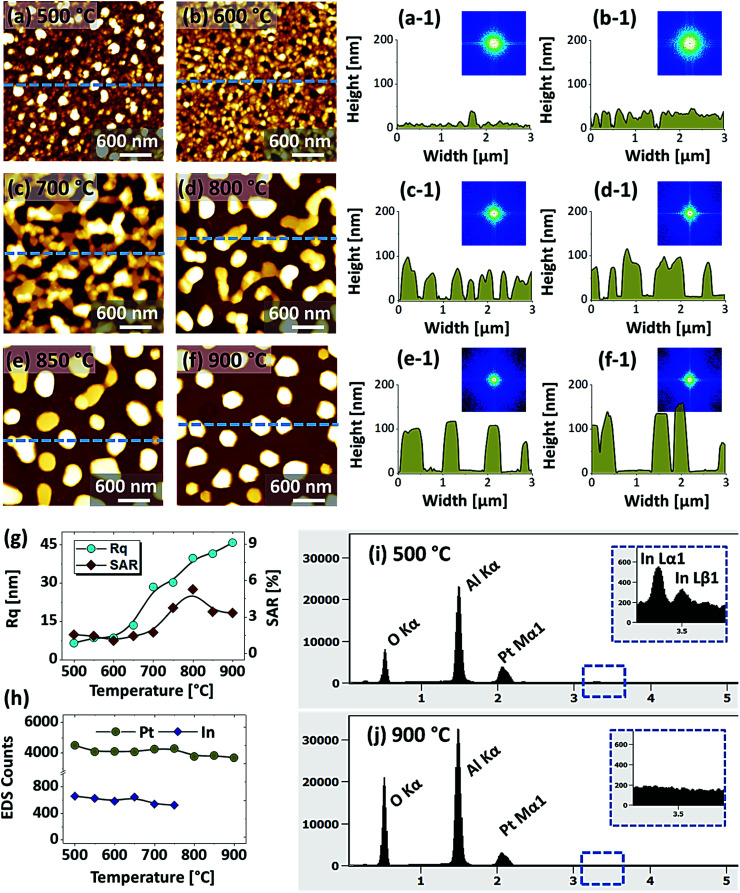
Evolution of Pt nanoclusters and NPs from the In_75 nm_/Pt_25 nm_ bilayers, annealed between 500 and 900 °C for 450 s. (a)–(f) AFM top-views of 3 × 3 μm^2^. (a-1)–(f-1) Cross-sectional line profiles. Insets show the FFT power spectra of the AFM images. (g) Summary plots of the *R*_q_ and SAR. (h) EDS counts of Pt Mα1 at 2.047 keV and In Lα1 at 3.287 keV with respect to the temperature. (i)–(j) Corresponding EDS spectra of the samples annealed at 500 and 900 °C respectively.

Fig. 3 shows the fabrication of Pt NPs with the In_75 nm_/Pt_25 nm_ bilayers by the variation of annealing temperature at an identical growth condition; *i.e.* between 600 and 900 °C for 450 s. A sharp distinction in terms of the size, configuration and areal density of Pt NPs was observed from the previous set. In this case, the total bilayer thickness was 100 nm, which consisted of 75 nm In and 25 nm Pt layer in a sequence. Due to the increased thickness of bilayer film, the overall dewetting process was significantly altered, which resulted in the gradual formation of grain-voids, irregular nanoclusters and large hexagonal Pt NPs at increased temperature. As discussed, the dewetting of thin film strongly depends upon the initial film thickness as well as the individual thickness of constituent elements in the case of metallic bilayers.^43^ Generally, thicker film can induce additional stability against the dewetting process due to which high temperature is required to obtain definite and isolated NPs.^44^ In this case, the overall thickness was increased but the ratio of In was three times higher than Pt and thus the dewetting process can be significantly boosted with temperature. This can be correlated to the increased influence of In component due to which the In–Pt inter-mixing, alloy formation and rate of In evaporation can be enhanced simultaneously. On the other hand, for the increased film thickness the nucleation of voids can be hindered, however, once formed, the voids can be grown significantly large before the formation of isolated NPs.^45^ Consequently, the resulting NPs can become larger in size and isolated from each other. This set of samples clearly demonstrated the formation of Pt NPs with larger size and increased spacing as compared to the previous set. In specific, as shown in Fig. 3(a) at 500 °C, the dewetting process was initiated with the formation of tiny pinholes and overgrown small NPs of ∼30 nm height and ∼150 nm diameter. Due to the enhanced diffusion of atoms and sublimation of In, the void formation and growth occurred between 500 and 700 °C, which resulted in the evolution of connected Pt nanoclusters. The voids were expanded up to several hundreds of nanometers and the nanoclusters height reached ∼60 nm as displayed by the corresponding line profiles in Fig. 3(b-1)–(c-1). The connected Pt nanoclusters were broken at 800 °C due to the Rayleigh-like instability,^46^ which resulted in the formation of isolated-irregular Pt NPs of ∼100 nm height and ∼500 nm width as shown in Fig. 3(c). Consequently, the irregular Pt NPs were gradually developed into the regular shape such as the truncated hexagonal and semi-spherical when the temperature was increased between 850 and 900 °C as shown in Fig. 3(e)–(f). Along with the increased temperature, the surface height fluctuation and uniformity of Pt NPs was improved as denoted by the reduced FFT pattern size. The shape of Pt NPs can be determined by the equilibrium energy distribution between surface and interface, contact angle as well as the crystallographic lattice structure of *c*-plane sapphire.^33,34^ Nevertheless, along with the enhanced surface diffusion of atoms at increased temperature, the NPs tend to gain the equilibrium configuration with the lowest possible surface energy and become thermally stable.^47^ The average height and width of Pt NPs were about 140 and 500 nm respectively, which is significantly larger than the Pt NPs obtained in the previous set. Furthermore, the areal density of NPs was reduced in this set, which was compensated by the larger size. The SEM images are provided in Fig. S6† to show the overall large-scale surface morphology of Pt NPs. In addition, the *R*_q_ and SAR were extracted from the large-scale AFM images and summarized with respect to the annealing temperature as shown in Fig. 3(g). From the summary plots, the *R*_q_ was gradually increased along with the temperature, which implies that the average surface height was increased by the evolution of Pt NPs. On the other hand, the SAR was increased up to 800 °C and decreased at higher temperature, which can be due to the density reduction. Moreover, the EDS peak counts are summarized in Fig. 5(h) and the EDS spectra of samples annealed at 500 and 900 °C are shown in Fig. 3(i) and (j) respectively. From the summary plot of EDS counts, the Pt counts were found to be consistent whereas In counts were sharply decreased above 700 °C. Unlike to the previous sets, the amount of residual In in this set was noteworthy up to 750 °C and then vanished at higher temperatures. This again clearly suggested the evolution of nearly pure Pt NPs can require either high annealing temperature or longer annealing duration.

**Fig. 4:**
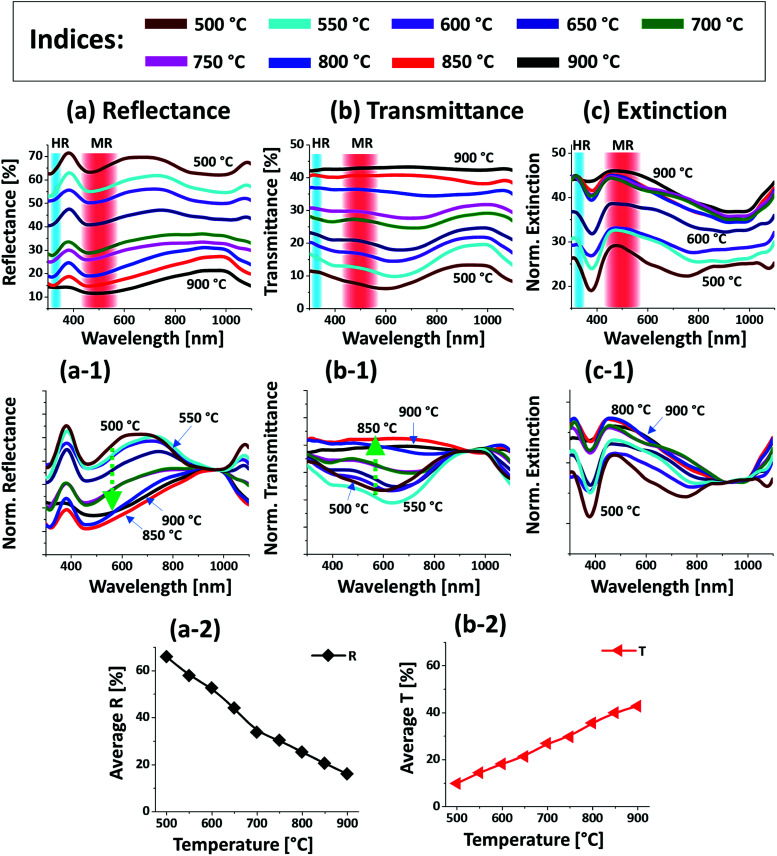
Optical characteristics of the Pt nanostructures fabricated between 500 and 900 °C with the In_75 nm_/Pt_25 nm_ bilayers. (a)–(c) UV-VIS-NIR reflectance (*R*), transmittance (*T*) and extinction spectra acquired from the measurement. The formation of higher-order resonance (HR) and multipolar resonance (MR) modes are indicated by the color bands in (a)–(c). (a-1) Normalized reflectance spectra. (b-1) Normalized transmittance spectra. (c-1) Normalized extinction spectra. (a-2) & (b-2) Summary plots of the average *R* and *T*.

**Fig. 5:**
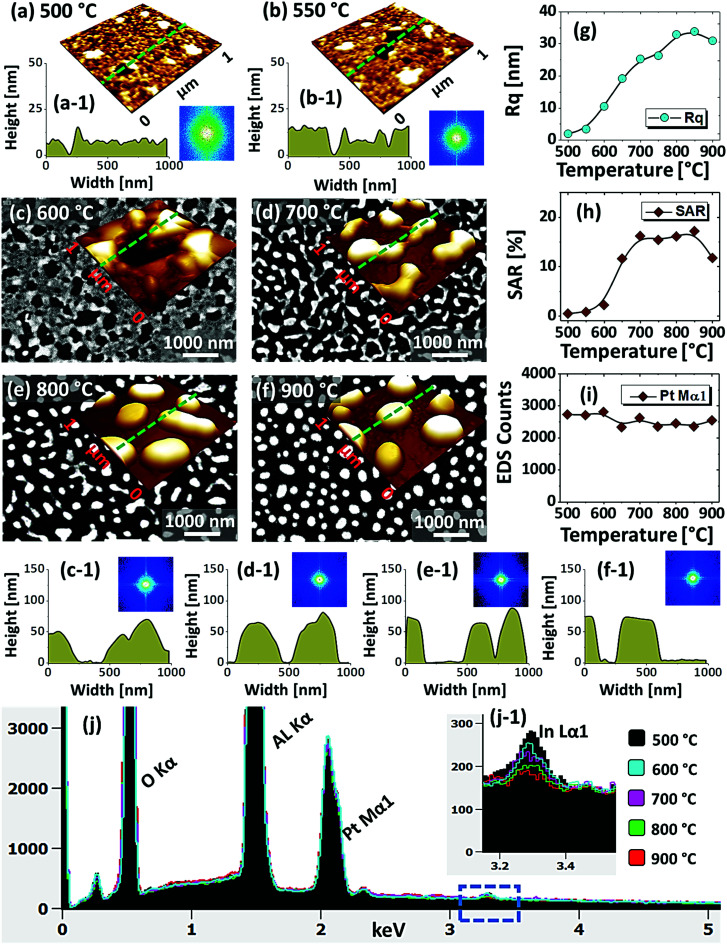
Formation of various configurations of Pt nanostructures by the dewetting of In_20 nm_/Pt_20 nm_ bilayers between 500 and 900 °C for 450 s. (a)–(b) AFM 3D side-views (1 × 1 μm^2^) of low temperature samples. (a-1)–(b-1) Corresponding line profiles and insets show FFT power spectra. (c)–(f) Scanning electron microscope (SEM) images of Pt nanostructures above 600 °C. Insets shows the corresponding AFM 3D side-views of 1 × 1 μm^2^. (c-1)–(f-1) Cross-sectional line profiles extracted from AFM side-views. (g)–(i) Summary plots of *R*_q_ and SAR and EDS count with respect to temperature. (j) EDS spectra of the Pt NPs. (j-1) Enlarge regime between 3.2 and 3.4 eV to show the In Lα1 peak.

Fig. 4 presents the optical properties of various large Pt NPs on sapphire based on the UV-VIS-NIR reflectance, transmittance and extinction spectra. The large size Pt NPs clearly demonstrated distinct optical properties in comparison with the small Pt NPs in the previous set. In specific, the reflectance spectra in Fig. 4(a) exhibited dips in the UV region at ∼320 nm and in the visible region at ∼500 nm. Since the average size of isolated NPs in this set was greater than 500 nm, the reflectance dips in the UV and visible region can be contributed by the higher order (HR) and multipolar (MR), *i.e.* dipolar and quadrupolar, resonance modes respectively as indicated in Fig. 4(a).^37^ In addition, a broad shoulder was observed in the visible region between 600 and 700 nm, which can be correlated to the backscattering effect of the Pt NPs. In fact, the low temperature samples up to 650 °C had small surface features of 150 nm in width and 30 nm in height and large number of voids, which can be perceived to act as small NPs causing strong backscattering effect as seen in the previous case. By comparing with the previous set, the visible regime shoulder was found to be red-shifted as the Pt NPs in this set were normally larger.^40^ The backscattering effect was gradually attenuated, and the absorption was enhanced in the UV-visible region with the increased size and definite shape of Pt NPs at higher temperature, which can also be clearly observed in normalized reflectance spectra in Fig. 4(a-1).^35^ The transmittance spectra in Fig. 4(b) also showed a distinct behavior from the previous set, in which a visible dip at ∼700 nm and NIR shoulder at ∼950 nm were observed for the connected Pt nanoclusters at low annealing temperature. With the evolution of isolated and larger Pt NPs, the visible region was gradually increased yielding flatter spectra as shown by the normalized transmittance spectra in Fig. 4(b-1). This can be correlated to the increased forward scattering effect of the large size Pt NPs.^48^ Both the reflectance and transmittance spectra agree well that the larger Pt NPs showed higher forward scattering; *i.e.* smaller NPs showed high backward scattering as in the previous case. Therefore, in the case of large Pt NPs the multipolar resonance can be mainly contributed by the quadrupolar resonance with the minor effect of dipolar resonance mode.^37^ Furthermore, the overall LSPR effect of large Pt NPs is demonstrated by the extinction spectra in Fig. 2(c). It generally showed a wide peak in the visible region and a minor peak in the UV region corresponding to the multipolar and higher order resonance of large Pt NPs as discussed. The overall extinction can be enhanced as the well-developed isolated Pt NPs can exhibit higher absorption and/or scattering than 2-D layer due to the LSPR effect. Further, the LSPR enhancement by the evolution of isolated, definite and large Pt NPs can be clearly seen in Fig. 4(c-1) with the normalized extinction spectra. As seen, the UV and VIS resonance peaks were gradually intensified with the dewetting of isolated NPs and the multipolar resonance peak in the visible region appeared to be generally wider as compared to the previous set, which can be correlated to the wider size distribution of Pt NPs as well as superposition of various resonance modes in the large Pt NPs.^37,41^ In addition, the average surface reflectance and transmittance are summarized in Fig. 4(a-2) and (b-2) respectively, in which the *R* & *T* showed the opposite trend with the gradually reduced surface coverage of Pt NPs as in the previous set.

Fig. 5 shows the fabrication of large semi-spherical Pt NPs with the In_20 nm_/Pt_20 nm_ bilayer at an equivalent growth condition as in the previous sets. Although the overall dewetting trend was quite similar with the previous set, the configuration and size of the Pt NPs were clearly varied. From the theory of dewetting kinetics, the dewetting process strongly depends on the initial film thickness, which allows to alter the surface configurations, size distribution and density at an identical growth condition^43^ and the individual thickness of constituent element also affects the dewetting process in the case of metallic bilayer films as mentioned. In this set, the overall thickness of In/Pt bilayer was in between the previous two sets. Therefore, the dewetting degree can be altered to generate distinct surface configuration of the Pt NPs. When sufficient temperature is provided, the overall atomic diffusion, intermixing between In and Pt atoms and In–Pt alloying can be enhanced as discussed. This gives a rise to the rapid dewetting process, resulting in the formation of definite nanostructures at relatively lower temperature. Specifically, the large voids of ∼100 nm in width were observed at even 500 °C as shown in Fig. 5(a), which is a distinction from the previous result at the same temperature. This clearly indicated that the void nucleation and growth occurred at lower temperature in this set likely due to lower Pt thickness. Although the thickness of In was much lower here, the Pt thickness seem to large affect the dewetting kinetics especially at the lower temperature. The voids were gradually coalesced and expanded up to ∼300 nm in width and grains of ∼50 nm height were also observed up to 600 °C in Fig. 5(c) and (c-1). By increasing the temperature, the diffusing atoms can be agglomerated around the grains and dewetting front or void edges can be expanded due to the surface capillary forces.^45^ These processes may lead to the coalescence of voids and growth of Pt nanoclusters, which can be driven by the reduction of surface and interface energy.^49^ At 700 °C, the average surface coverage was significantly reduced as the connected Pt nanoclusters were developed with ∼60 nm average height and ∼280 nm average width as shown in Fig. 5(d) and (d-1). A drastic surface morphology transition was also observed at 800 °C when the nanoclusters were broken into the isolated-irregular Pt NPs as shown in Fig. 5(e) and (e-1). This fact can be correlated to the surface energy anisotropy and Rayleigh-like instability of large nanoclusters as discussed.^46^ The average height and diameter of isolated NPs were further increased to ∼80 and 300 nm respectively due to the accumulation of atoms with the reduced surface energy. Finally, the isolated-irregular NPs were transformed into the semi-spherical NPs with ∼75 nm average height and ∼350 nm average width at 900 °C. The formation of semi-spherical configuration of Pt NPs can be due to the isotropic surface energy distribution.^33^ At the same time, the top surface of Pt NPs became flatter as shown in Fig. 5(f-1) likely due to the formation of lower surface energy plane.^35,50^ Similar to the pervious sets, the uniformity of Pt NPs was improved with temperature as indicated by the FFT patterns. From the results in the previous and this sets, it clearly evidenced that the definite surface morphology of Pt NPs with different configurations can be realized by the appropriate control of In and Pt thickness and ratio. However, the hexagonal shape of Pt NPs was not observed in this set, which can be due to the low In ratio in the In/Pt bilayers. The evolution of *R*_q_ and SAR for this series of samples generally also showed an increasing trend along with the development of Pt nanostructures as shown in Fig. 5(g) and (h) respectively. The *R*_q_ was gradually increased up to 800 °C, whereas decreased at 900 °C due to the height decrement of Pt NPs by the formation of a flat top. The SAR was sharply increased up to 700 °C due to the rapid evolution of nanoclusters and then, remained almost similar up to 800 °C as the average surface area can be similar because the size and density of Pt NPs can compensate each other. However, it was sharply decreased at 900 °C due to the large reduction of surface coverage by the formation of isolated Pt NPs. In the summary counts of EDS peaks and spectra in Fig. 5(i) and (j), the Pt Mα1 intensity was almost similar for all samples, suggesting the identical amount of Pt regardless of various surface morphologies. In addition, a weak In Lα1 peak was detected whose intensity was gradually decreased with temperature as shown in Fig. 5(j-1). This again confirmed that the evolution of Pt NPs was occurred due to the concurrent effect of dewetting and sublimation of In atoms. The intensity of In peak was almost equal to the background above 800 °C.

**Fig. 6:**
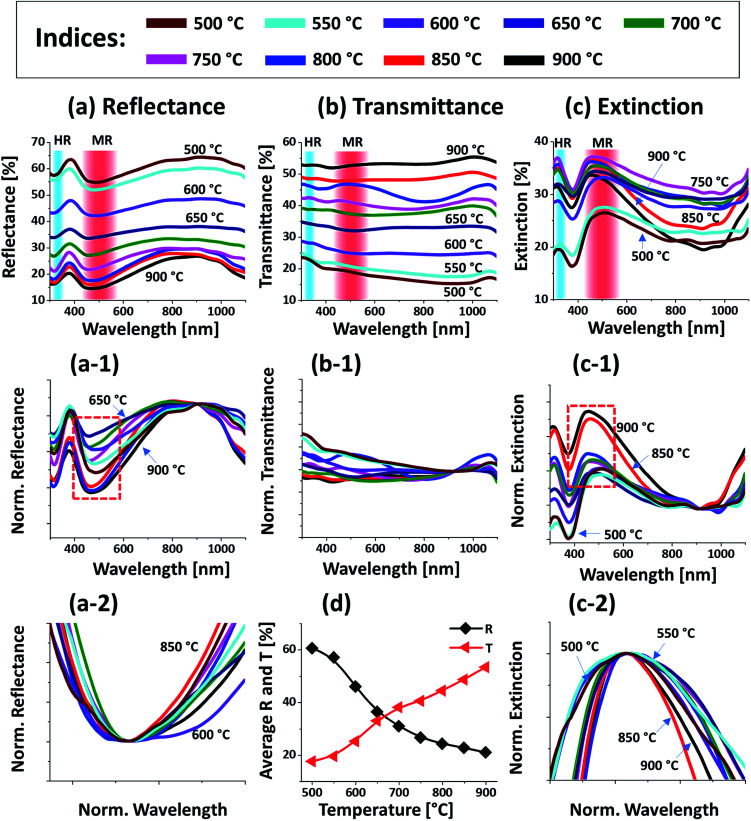
Optical characteristic of the Pt NPs fabricated by the annealing of In_20 nm_/Pt_20 nm_ bilayers at 500 to 900 °C for 450 s. (a)–(c) Reflectance, transmittance and extinction spectra. (a-1) Normalized reflectance spectra. (a-2) Enlarged dip region of reflectance spectra, boxed region in (a-1). (b-1) Normalized transmittance spectra. (c-1) Normalized extinction spectra. (c-2) Enlarged extinction peaks, boxed region in (c-1). (d) Summary of average *R* and *T*.

Fig. 6 present the optical properties of Pt NPs fabricated with the In_20 nm_/Pt_20 nm_ bilayers between 500 and 900 °C. In this set, the obtained Pt NPs had average size slightly smaller than the previous two sets whereas the NPs size and configuration were varied significantly. As a result, the resulting optical characteristics clearly reflected the difference from the previous sets. As shown in Fig. 6(a), the reflectance spectra generally exhibited a dip in the UV at ∼320 nm and a wider dip in the visible region at ∼500 nm, which can be correlated to the higher order and multipolar resonance modes of large Pt NPs respectively as discussed.^37,41^ Furthermore, the trend of resonance dips evolution with different shape and size of Pt NPs is shown by the normalized reflectance spectra in Fig. 6(a-1). It was found that the UV and VIS region dips were gradually increased, indicating gradually enhanced absorption due to the much stronger LSPR effect of the isolated and larger Pt NPs at high temperature. In contrast to the previous set, the low annealing temperature samples below 650 °C didn't show backscattering effect^37,38^ and this can be due to the larger size of the nanoclusters and voids resulted at low annealing temperature in this set. However, after the formation of isolated NPs above 700 °C, both sets showed similar spectral response with enhanced forward scattering and absorption in the visible region. Furthermore, the reflectance dips showed a narrowing behavior as displayed in Fig. 6(a-2) along with the improved size and shape uniformity of Pt NPs. From the transmittance spectra in Fig. 6(b) and (b-1), minor shoulders in the UV and visible region were observed, which can be correlated to the forward scattering effect by the large size Pt NPs around the resonance wavelength as discussed.^37,38^ In fact, the transmittance spectra should also exhibit absorption dips at the resonance wavelength such as in Fig. 2(b), however, due to the forward scattering of large Pt NPs the dips were buried in the spectra. Further, the LSPR excitation of Pt NPs is demonstrated by the extinction spectra in Fig. 6(c), which exhibits a strong multipolar resonance band in the visible region and a minor higher order peak in the UV region.^37^ As shown in the normalized extinction spectra in Fig. 6(c-1), the LSPR peaks were gradually increased indicating enhanced absorption with the formation of isolated and larger Pt NPs along with the dewetting. It was also noticed that the LSPR band gradually became narrower as displayed in Fig. 6(c-2) due to the improved uniformity of Pt NPs at higher temperature.^40^ In case of average reflectance, it was gradually decreased with the reduced surface coverage of Pt NPs whereas the average transmittance was increased as shown in Fig. 6(d).

## Conclusions

In conclusion, this work demonstrated the fabrication of well-structured, isolated and regular Pt NPs through the enhanced thermal dewetting process facilitated by a sacrificial In layer. The enhancement in the dewetting process was attributed to the high surface diffusivity, low surface energy, low melting point and sublimation temperature of the intermediate In layer. During annealing, the intermixing of Pt and In atoms and formation of In–Pt alloy system contributed to the enhanced global diffusion of atoms. Then, along with the dewetting of alloy nanostructures, the In atoms sublimated and finally pure Pt NPs were fabricated. Based on the control of bilayer thickness, the varieties of Pt NPs were fabricated. As compared to the Pt NPs fabricated using the pure Pt film, a significant improvement in the size, spacing and shape of Pt NPs was accomplished in this work. For instance, the largely isolated hexagonal Pt NPs with the In_75 nm_/Pt_25 nm_, moderately gapped semispherical Pt NPs with the In_20 nm_/Pt_20 nm_ and compactly organized small spherical and hexagonal Pt NPs with the In_2.5 nm_/Pt_7.5 nm_ bilayer were demonstrated. Furthermore, the optical properties exhibited a strong sensitivity to the variation of size, spacing and configuration of Pt NPs due to which, the LSPR band was tuned over ∼400–700 nm. The LSPR peak position was generally blue shifted by the increased spacing and decreased size. And, the LSPR peak width was narrowed by the formation of regular and uniform Pt NPs. Finally, this study can be utilized to produce diverse Pt nanostructures and their LSPR band can be tuned over a broad range for the applications in plasmonic, catalytic and optoelectronic applications.

## Conflicts of interest

There are no conflicts to declare.

## Supplementary Material

RA-009-C8RA09049A-s001
