# Supplementary material for: Improved control on the morphology and LSPR properties of plasmonic Pt NPs through enhanced solid state dewetting by using a sacrificial indium layer
Source: RSC Adv. 2019 Jan 17;9(4):2231–43. doi: 10.1039/c8ra09049a (PMC9059813; doi:10.1039/c8ra09049a)
Supplement: RA-009-C8RA09049A-s001 [file RA-009-C8RA09049A-s001.pdf]

Electronic Supplementary Material (ESI) for RSC Advances.
This journal is © The Royal Society of Chemistry 2019

Electronic supplementary information (ESI)

Improved control on the morphology and LSPR properties of plasmonic Pt NPs through the enhanced solid state dewetting by using a sacrificial indium layer

Sundar Kunwar, Mao Sui, Puran Pandey, Zenan Gu, Sanchaya Pandit and Jihoon Lee*

Department of Electronic Engineering, College of Electronics and Information, Kwangwoon University, Nowon-gu Seoul 01897, South Korea. *Correspondence e-mail: jihoonlee@kw.ac.kr

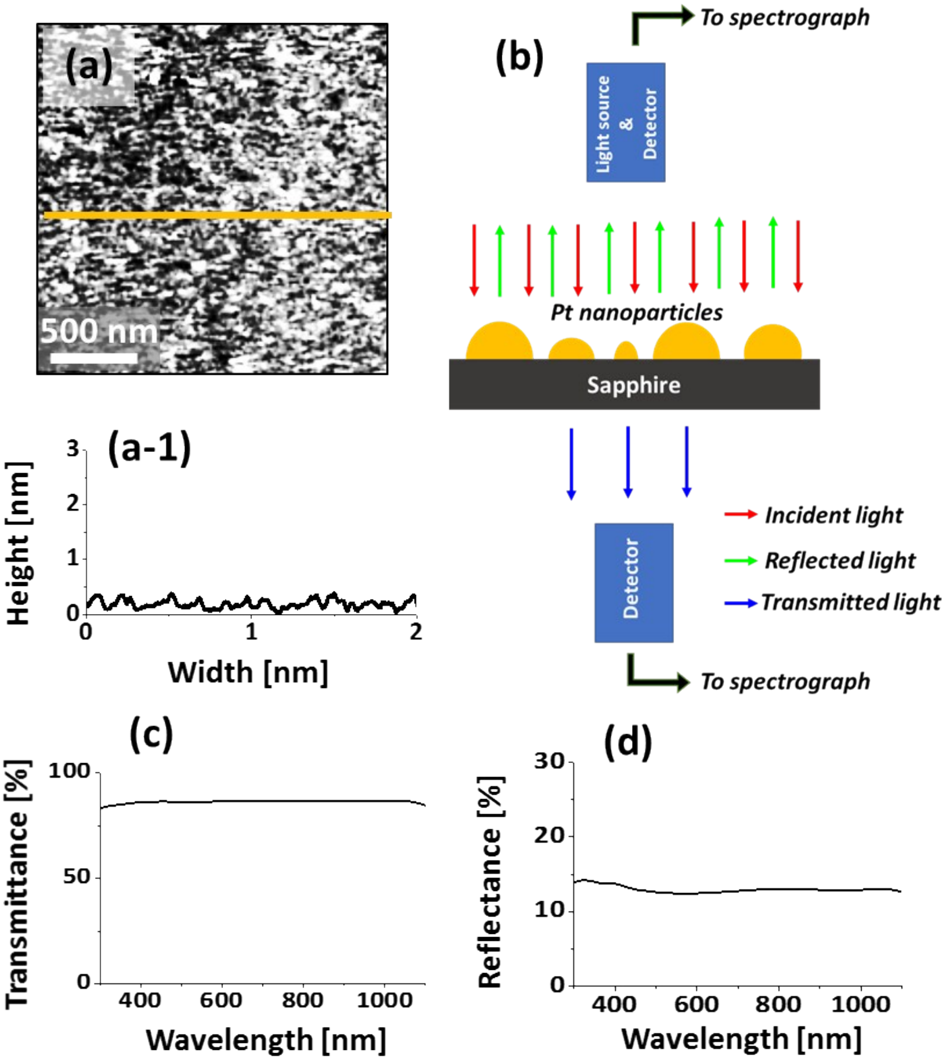

Figure S1: (a) AFM top-view of bare sapphire (0001). (a-1) Cross-sectional line profile obtained from the AFM image. (b) Schematic representation of experimental setup for the transmittance and reflectance measurements. (c) - (d) Transmittacne and reflectance spectra of bare sapphire (0001).

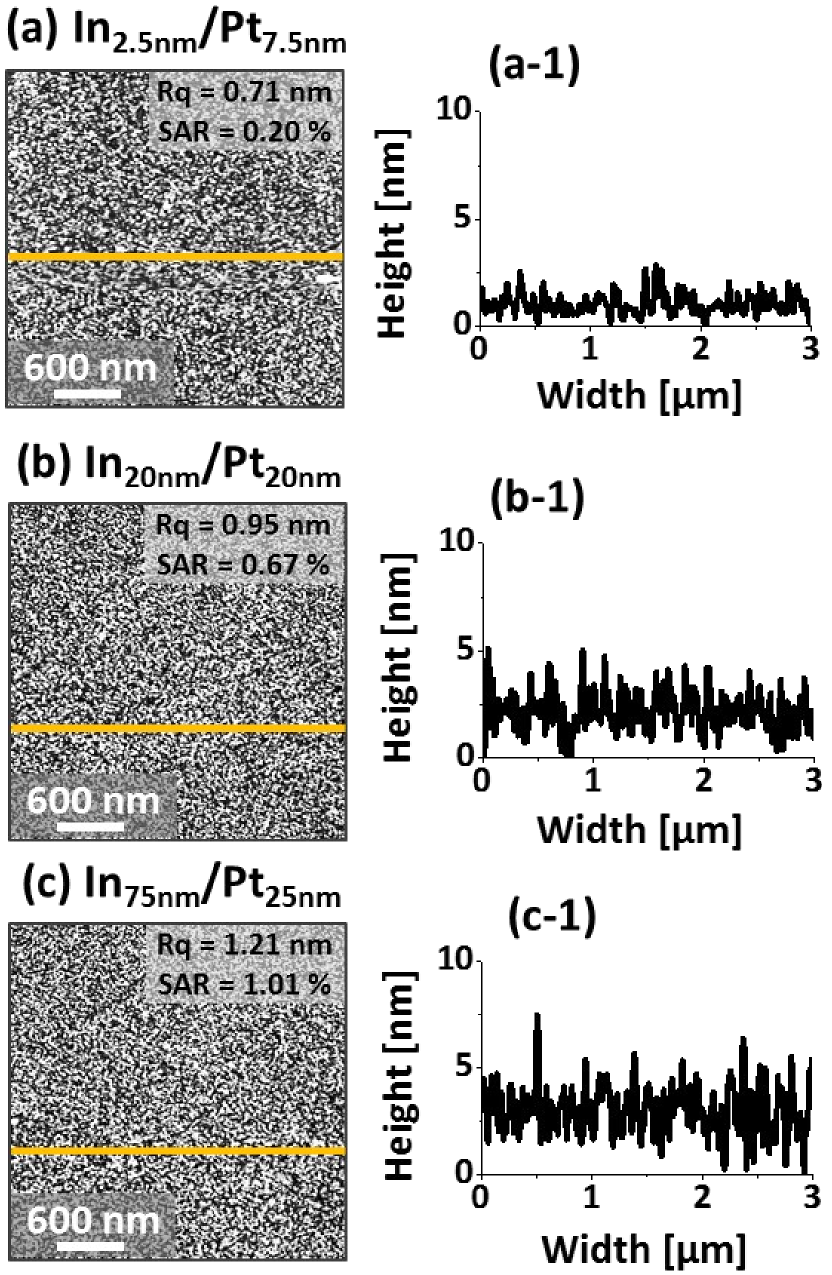

Figure S2: Surface morphology of In/Pt bilayer deposited on sapphire (0001) with various thickness as labelled. (a) – (c) AFM top-views 3 × 3 µm^2. (a-1) – (c-1) Corresponding cross-sectional line profiles of the AFM top-views. The RMS roughness (Rq) and surface area ratio (SAR) showed increasing values with higher thickness of In/Pt bilayer.

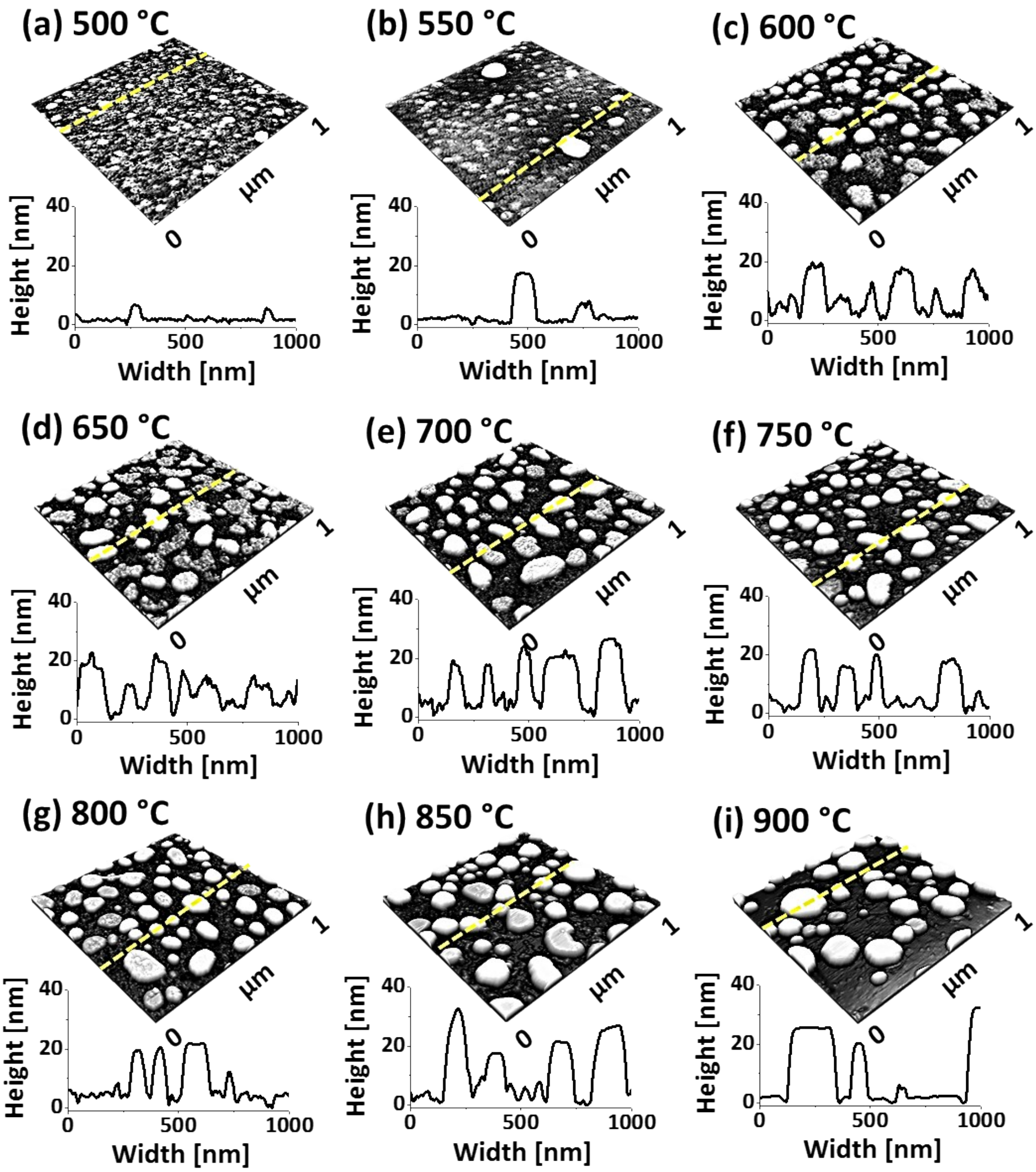

Figure S3: Evolution of Pt nanostructures from the $In_{2.5nm}/Pt_{7.5nm}$ bilayer annealed between 500 and 900 °C for 450 s. (a) – (i) AFM side-views of 1 × 1 μm^2 along with the cross-sectional line profiles.

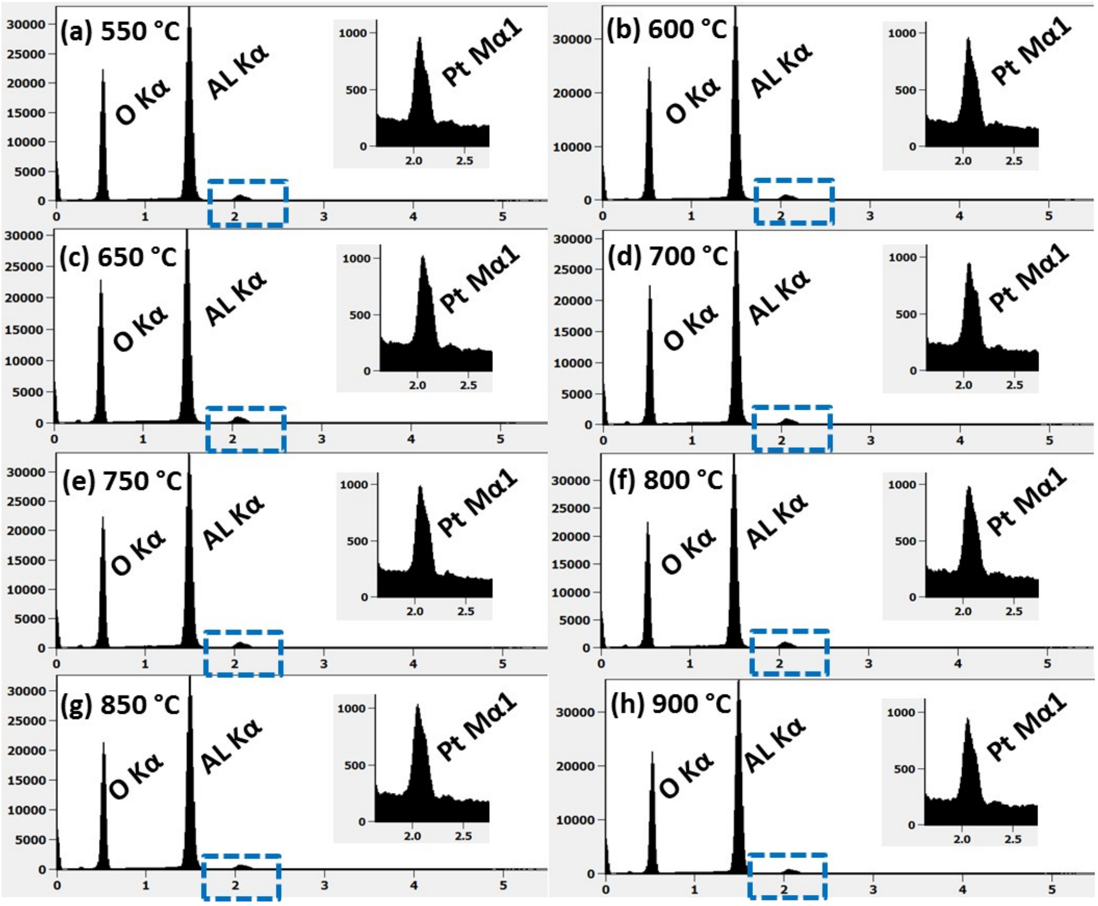

Figure S4: (a) – (h) EDS spectra of the Pt nanostructures on sapphire fabricated between 550 and 900 °C with $In_{2.5nm}/Pt_{7.5nm}$ bilayer. O and Al peaks correspond to the substrate and Pt peak corresponds to the nanostructures. The Pt peak intensity is consistent for all samples.

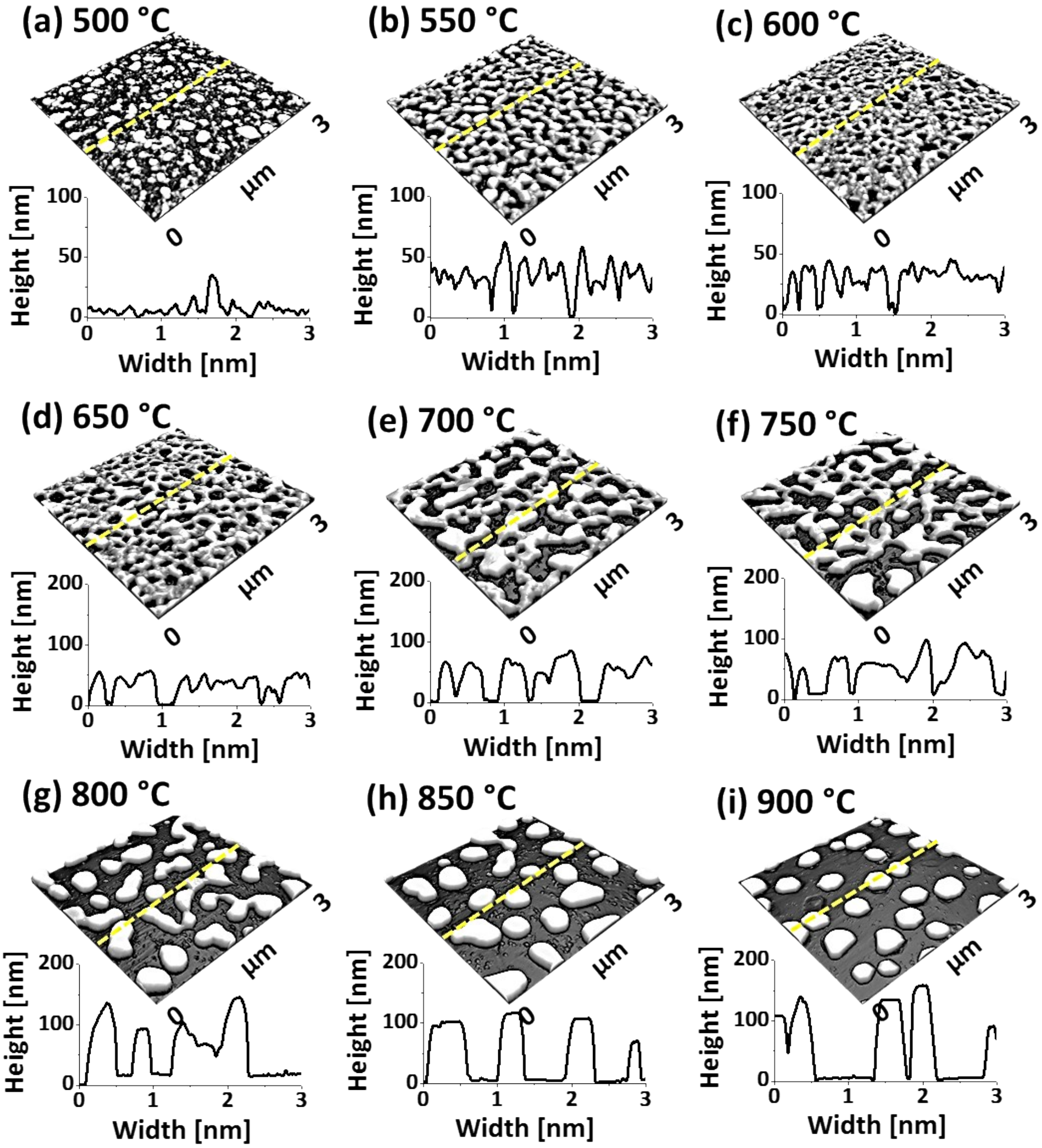

Figure S5: Evolution of Pt nanostructures from In_{75nm}/Pt_{25nm} bilayer by the annealing between 500 and 900 °C for 450 s. (a) – (i) AFM side-views of 3 × 3 μm^2 along with the cross-sectional line profiles.

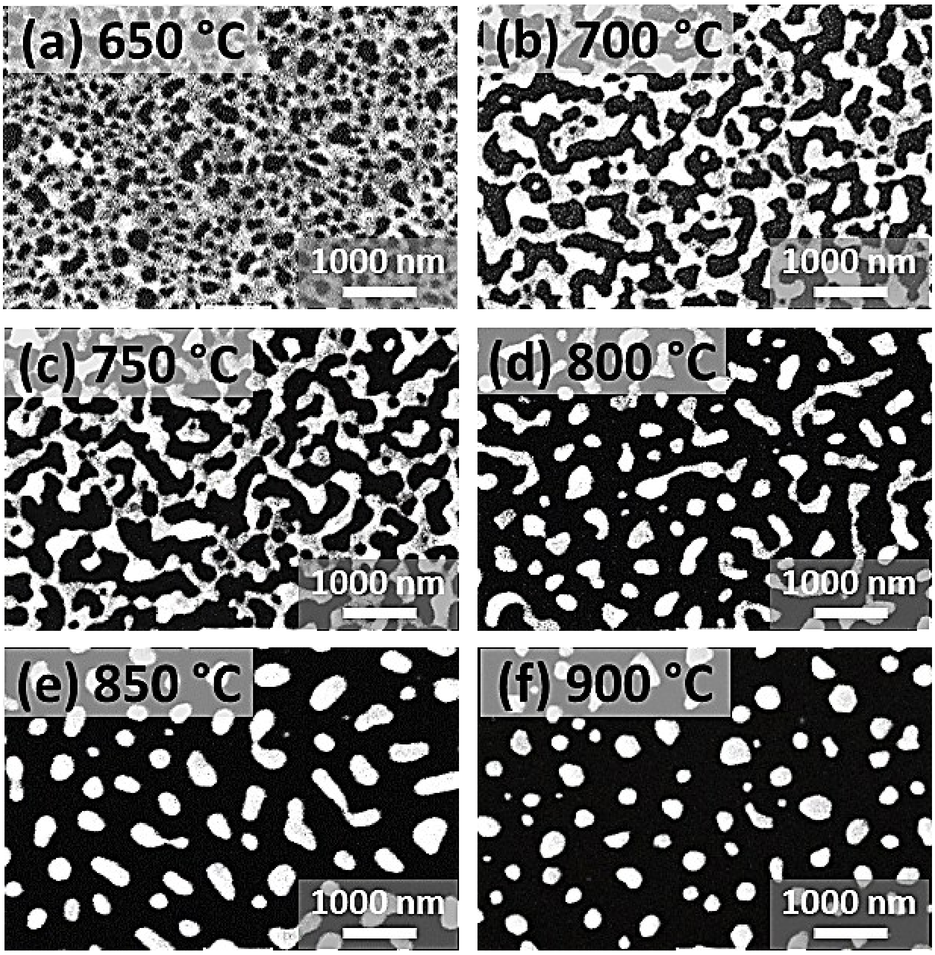

Figure S6: SEM images of the Pt nanostructures fabricated between 650 and 900 °C for 450 s with the In_{75nm}/Pt_{25nm} bilayers.

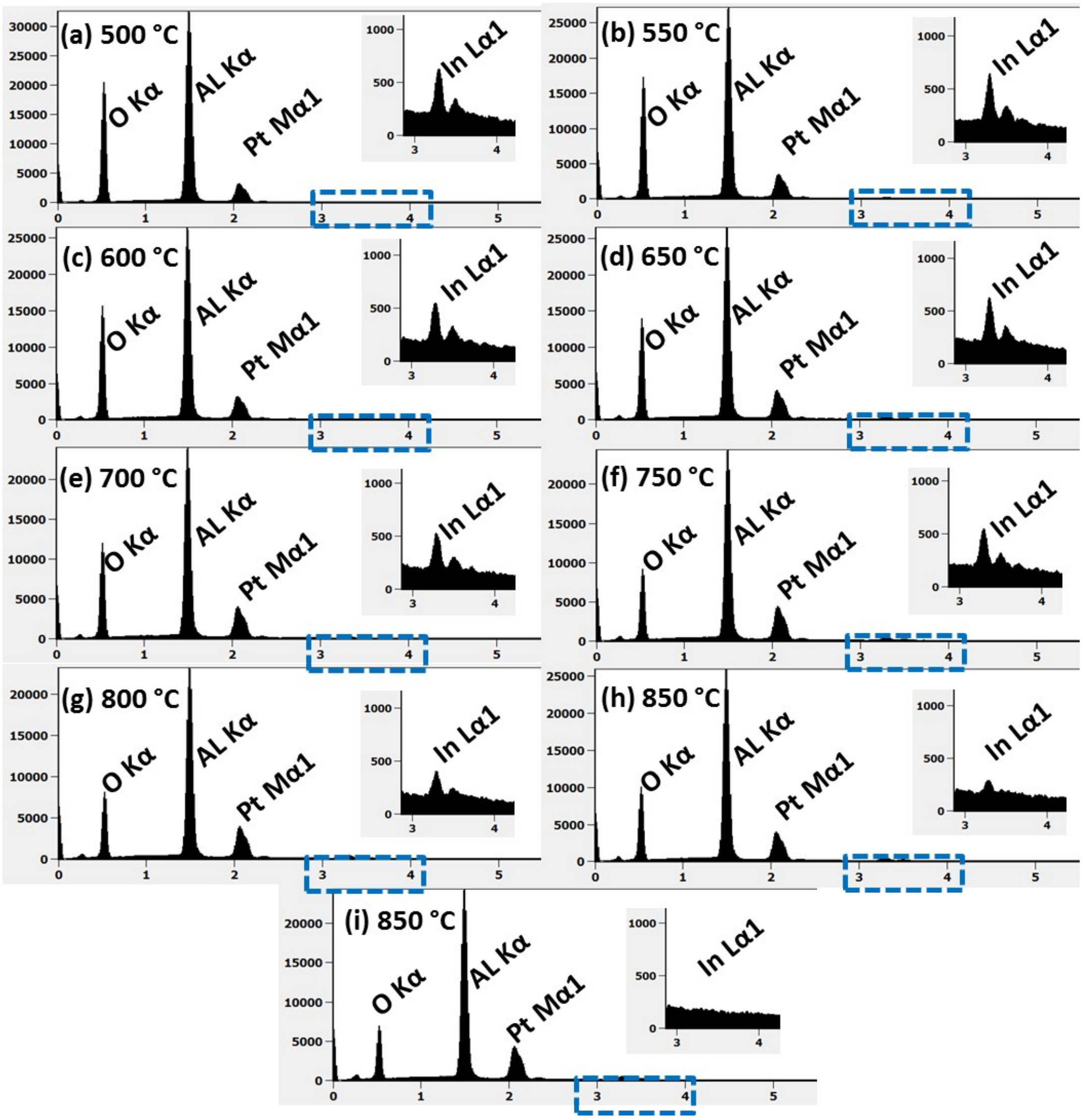

Figure S7: EDS spectra of the Pt nanostructures on sapphire fabricated between 550 and 900 °C with the In_{75nm}/Pt_{25nm} bilayers.

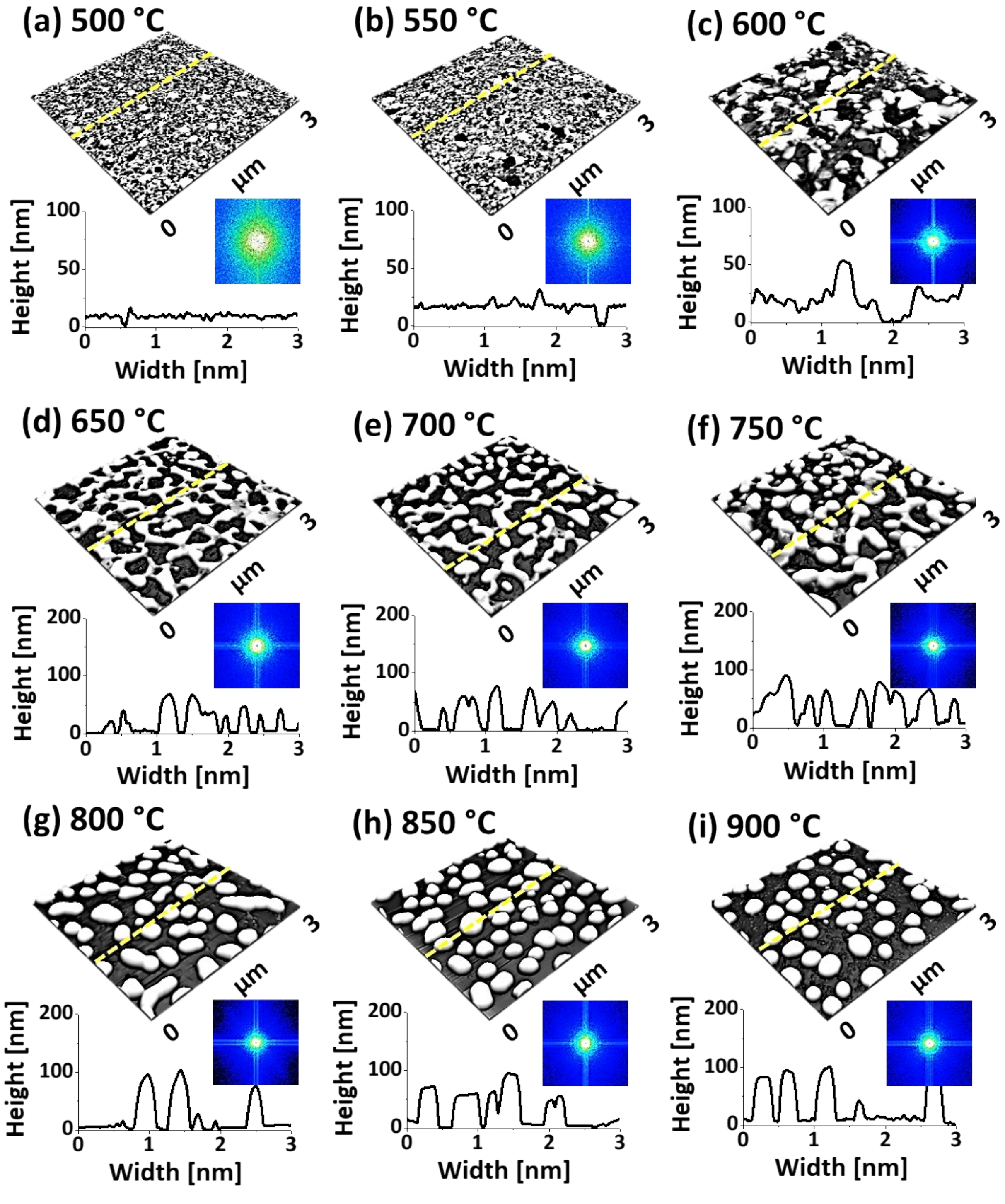

Figure S8: Formation of the Pt nanostructures on sapphire (0001) by the dewetting of the In_{20nm}/Pt_{20nm} bilayers based on the annealing between 500 and 900 °C for 450 s. (a) – (i) AFM side-views of 3 × 3 μm^2. Insets show the cross-sectional line profiles and Fourier filter transform (FFT) power spectra of AFM images.

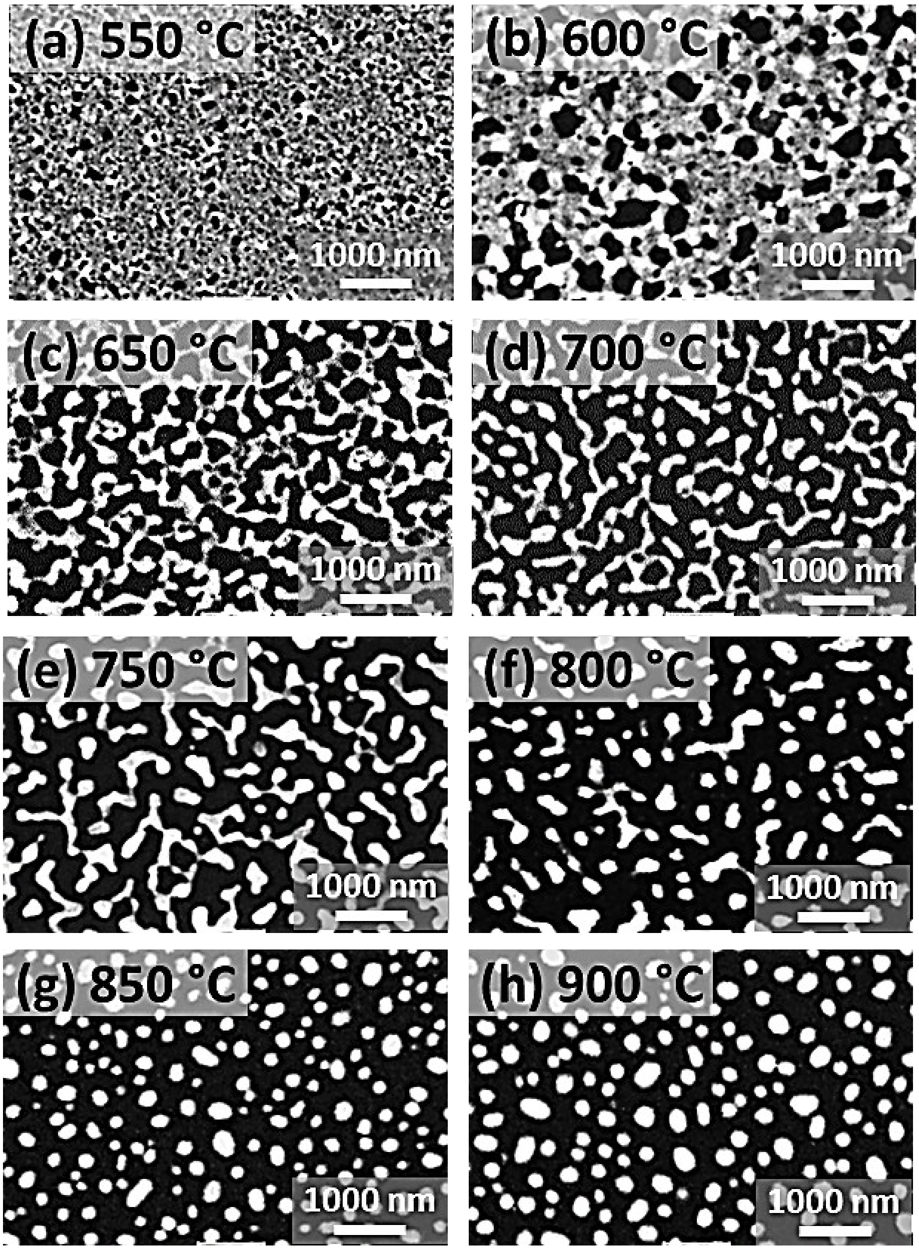

Figure S9: SEM images of the Pt nanostructures fabricated between 550 and 900 °C for 450 s with In_{20nm}/Pt_{20nm} bilayer.

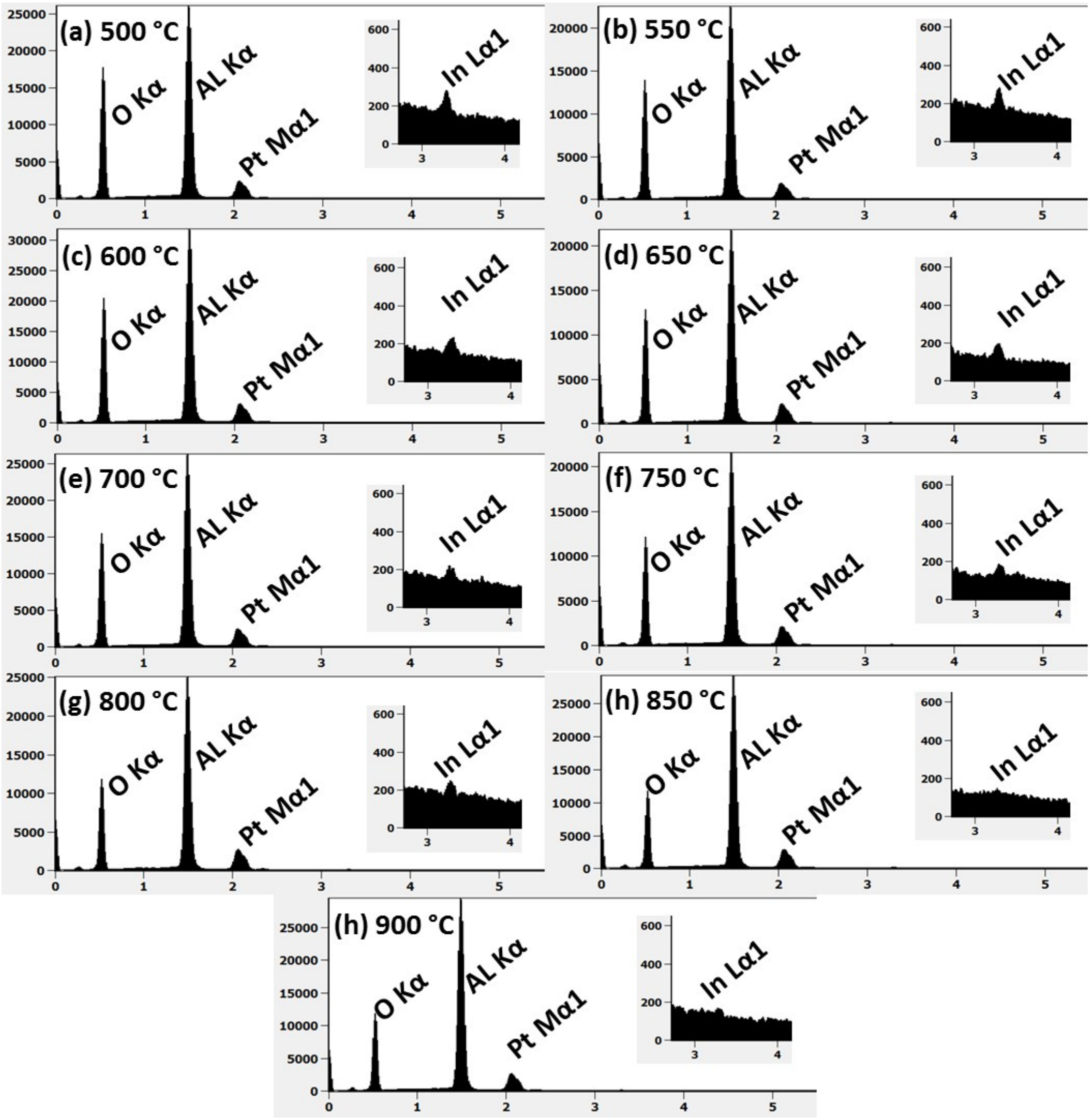

Figure S10: EDS spectra of the Pt nanostructures on sapphire fabricated between 500 and 900 °C with the In_{20nm}/Pt_{20nm} bilayer. O and Al peaks corresponds to the substrate and Pt peaks to the nanostructures. The Pt peak intensity is consistent for all samples. Insets show the In peak.

Table S1: Summary of RMS roughness (Rq) and surface area ratio (SAR) of the Pt nanostructures fabricated between 500 and 900 C for 450 s with different thickness of In and Pt in the bilayers as labelled.

Temperature [°C]	$In_{2.5nm}/Pt_{7.5nm}$		In_{20nm}/Pt_{20nm}		In_{75nm}/Pt_{25nm}	
	**Rq [nm]**	**SAR [%]**	**Rq [nm]**	**SAR [%]**	**Rq [nm]**	**SAR [%]**
**500**	2.66	0.551	1.85	0.4	6.32	1.5289
**550**	2.26	0.6212	3.29	0.73	13.31	8.7347
**600**	5.22	4.2989	10.39	2.1374	8.41	4.401
**650**	5.36	4.8427	19.09	11.5355	13.32	7.1106
**700**	6.7	6.5583	25.21	16.1509	28.32	14.8781
**750**	5.71	5.0458	26.23	12.3632	30.1	14.9044
**800**	6.67	5.9541	32.83	12.3632	39.61	18.2117
**850**	8.17	7.5214	33.77	17.1355	41.38	14.1617
**900**	10.19	8.4658	30.89	11.6941	45.67	17.5146

Table S2: Summary of average reflectance (R) and transmittance (T) of the Pt nanostructures fabricated between 500 and 900 °C for 450 s with different thickness of In and Pt in the bilayers as label led.

Temperature [°C]	$In_{2.5nm}/Pt_{7.5nm}$		In_{20nm}/Pt_{20nm}		In_{75nm}/Pt_{25nm}	
	R [%]	T [%]	R [%]	T [%]	R [%]	T [%]
**500**	2.66	0.551	1.85	0.4	6.32	1.5289
**550**	2.26	0.6212	3.29	0.73	13.31	8.7347
**600**	5.22	4.2989	10.39	2.1374	8.41	4.401
**650**	5.36	4.8427	19.09	11.5355	13.32	7.1106
**700**	6.7	6.5583	25.21	16.1509	28.32	14.8781
**750**	5.71	5.0458	26.23	12.3632	30.1	14.9044
**800**	6.67	5.9541	32.83	12.3632	39.61	18.2117
**850**	8.17	7.5214	33.77	17.1355	41.38	14.1617
**900**	10.19	8.4658	30.89	11.6941	45.67	17.5146
